# Healthcare-associated infections and antimicrobial use in acute care hospitals in Greece, 2022; results of the third point prevalence survey

**DOI:** 10.1186/s13756-024-01367-8

**Published:** 2024-01-25

**Authors:** Konstantinos Palaiopanos, Dimitra Krystallaki, Kassiani Mellou, Petros Kotoulas, Christina-Anna Kavakioti, Styliani Vorre, Georgia Vertsioti, Maria Gkova, Antonios Maragkos, Kyriaki Tryfinopoulou, Dimitrios Paraskevis, Sotirios Tsiodras, Theoklis Zaoutis

**Affiliations:** 1 Directorate of Epidemiological Surveillance and Intervention for Infectious Diseases, National Public Health Organization (EODY), Athens, Greece; 2 Central Public Health Laboratory, National Public Health Organization, Athens, Greece; 3 Department of Hygiene, Epidemiology, and Medical Statistics, Medical School, National and Kapodistrian University of Athens, Athens, Greece; 4 4th Department of Internal Medicine , “Attikon” University Hospital, National and Kapodistrian University of Athens , Athens, Greece; 5 2nd Department of Pediatrics, “P. and A. Kyriakou” Children’s Hospital, National and Kapodistrian University of Athens, Athens, Greece

**Keywords:** Point prevalence survey, Infections, HAIs, Antimicrobials, Prescription, Stewardship, Inpatients, Prevalence, Greece

## Abstract

**Background:**

The burden of healthcare-associated infections (HAIs) and the extent of antimicrobial use (AU) are periodically recorded through Point Prevalence Surveys (PPS) in acute care hospitals coordinated by the European Centre for Disease Prevention and Control (ECDC). In previous PPSs, Greece demonstrated increased HAI and AU prevalence: 9% and 54.7% in 2011–2012, and 10% and 55.6% in 2016–2017, respectively. The 2022 PPS aimed to estimate HAIs and AU indicators among inpatients, especially amid the COVID-19 pandemic.

**Methods:**

A cross-sectional study was conducted in 50 hospitals during October-December 2022, in Greece. Patients admitted before 8.00 a.m. of the survey day were observed. Patients with at least one HAI or receiving at least one antimicrobial agent were included. Data were collected by hospital infection control teams. Hospital and ward-level variables were analysed.

**Results:**

From 9,707 inpatients, 1,175 had at least one HAI (12.1%), and 5,376 were receiving at least one antimicrobial (55.4%). Intensive care unit patients had the highest HAI (45.7%) and AU (71.3%) prevalence. Of the 1,408 recorded HAIs, lower respiratory tract (28.9%), bloodstream (20%), and urinary tract infections (13.1%) were the most common. Among 1,259 isolates, *Klebsiella* (20.5%) and *Acinetobacter* (12.8%) were most frequently identified. Resistance to first-level antibiotic markers was 69.3%. Among the 9,003 antimicrobials, piperacillin-tazobactam (10.9%), and meropenem (7.7%) were frequently prescribed. The ratio of broad-spectrum to narrow-spectrum antibiotics was 1.4. As defined by the 2021 WHO AWaRe (Access, Watch, Reserve) classification, restricted classes of Watch and Reserve agents comprised 76.7% of antibiotics. Usual indications were treatment of community-acquired infections (34.6%) and HAIs (22.9%). For surgical prophylaxis, cefoxitin was commonly used (20.2%), and typical courses (75.7%) lasted more than one day. HAI and AU prevalence were positively associated with bed occupancy (*p* = 0.027) and secondary hospitals (*p* = 0.014), respectively.

**Conclusions:**

The 2022 PPS highlighted the increasing trend of HAI prevalence and high AU prevalence in Greece, the emergence of difficult-to-treat pathogens, and the extensive use of broad-spectrum antimicrobials. Strengthening infection control and antimicrobial stewardship programs in hospital settings is essential.

**Supplementary Information:**

The online version contains supplementary material available at 10.1186/s13756-024-01367-8.

## Introduction

Healthcare settings can contribute to the spread of infections among staff and patients. In acute care hospitals, healthcare-associated infections (HAI) among patients can be particularly challenging to treat and control.

HAIs are a major public health challenge with a substantial impact on health systems and patients. The World Health Organization reports that, on average, seven out of every 100 patients in acute-care hospitals will acquire at least one HAI during their hospitalisation [1]. HAIs result in longer hospitalisation and increased healthcare costs, and are a significant cause of disability and decrease in quality of life. In the European Union and European Economic Area (EU/EEA) HAIs annually correspond to approximately 2.5 million disability-adjusted life years (DALYs) and represent the infections with the highest burden [2, 3].

Pathogens responsible for HAIs often exhibit multiple mechanisms of antimicrobial resistance (AMR). The main driver of AMR is excessive and indiscriminate antimicrobial use (AU) in humans, animals and plants. Antimicrobial stewardship programs (AMS) and infection prevention and control (IPC) measures can halt the development and spread of difficult-to-treat pathogens [4]. Countries have adopted various strategies to mitigate this problem [5]. A targeted approach needs surveillance of HAI and AU at the local level.

In Europe, the prevalence of HAIs and AU in hospitals is estimated through periodic Point Prevalence Surveys (PPSs) coordinated by the European Centre for Disease Prevention and Control (ECDC). In the last survey in 2016–2017, Greece was among the countries with highest prevalences [6].

In 2022, the National Public Health Organization (EODY) conducted a nationwide PPS. The objectives of the study were to (a) calculate HAI and AU prevalence, (b) report on the characteristics of HAI and AU in Greek hospitals, and (c) identify potential risk factors at the hospital level.

## Methods

### Study design

A cross-sectional study was conducted in 50 of the 126 hospitals of the Greek National Health Care System in 2022. Data collection took place in two predefined successive study periods, namely October to December for 48 hospitals, and March to April for two. The ECDC PPS protocol v.6.0 “Point prevalence survey of healthcare-associated infections and antimicrobial use in European acute care hospitals” in the unit-based (light) version was used, i.e., demographic data was only to be collected for patients with HAI and/or AU [7]. On the survey day, HAI and AU among inpatients were recorded.

A representative sample was drawn from the Greek national hospital register, using a systematic sampling design, as instructed in the protocol [7, 8]. In case of refusal of the first selected hospital, the next hospital on the list was selected. Participation was not incentivised and data from hospitals that were not selected were not included in this research.

Data were collected by the IPC team and the Infectious Diseases (ID) team of each hospital under EODY guidance. Electronic training material was provided to participating staff prior to data collection to support a standardized process and uniform recording. The provided material, composed by the EODY-based PPS team, included case-finding algorithms, recorded presentations, and the ECDC protocol, in original and translated version. The preparatory period lasted three weeks and involved daily guidance and feedback in both a synchronous and asynchronous manner. The time frame for data collection was a single day for each hospital ward and the study had to be completed in less than two to three weeks for all wards of each hospital [7].

### Study variables

Data collection was organized at the hospital, ward and patient level including factors potentially associated with HAI and AU prevalence. Hospital and ward characteristics were categorized as (a) general characteristics e.g., hospital size, hospital type (paediatric, secondary or tertiary hospital), (b) workload indicators e.g., bed occupancy in participating wards, percentage of surgical inpatients, healthcare staff to patient ratio, (c) indicators related to IPC and AMS e.g., number of full-time IPC team members, number of single rooms, and (d) metrics for COVID-19 e.g., proportion of currently hospitalised COVID-19 patients, vaccination coverage of healthcare workers.

Patients already hospitalised at the time of the survey that had been admitted before 8.00 am. of the same day were eligible to participate. Patients in emergency departments, daycare, residential care, and outpatients were excluded. The number of eligible patients provided denominator data for HAI and AU prevalence calculations. All participants were screened for the presence of HAI and AU of any indication and individual data (e.g., demographics, admission date) was collected for the subset of those on AU and/or with HAI to provide numerator data.

Active HAI cases on the day of the survey were recorded. For an infection to be considered active, signs and symptoms had to be present on the survey day or had to be previously present, and the patient had to be still under treatment for them. For an infection to be considered healthcare-associated, the onset had to be at least 48 h after admission. Exceptions were (a) infections in patients hospitalised in the previous 48 h in another hospital, (b) surgical site infections (SSI) occurring within 30 days of operation or 90 days if an implant was placed, (c) *C. difficile* infections occurring 28 days after hospital discharge, and (d) COVID-19 infections occurring after the third day of hospitalisation (indeterminate association), including the eighth (probable or definite healthcare-associated COVID-19). HAI data included infection type, onset date, presence of relevant device e.g., a urinary catheter in urinary tract infections (UTI) and possible place of exposure of HAI e.g., current hospital or long-term care facility (LTCF).

Microbiological results available on the survey day were collected. Each participating hospital had its routine practice and methodology for specimen collection, laboratory testing and antimicrobial susceptibility testing and reporting. The reporting of susceptibility results was based on the guidelines of the European Committee on Antimicrobial Susceptibility Testing (EUCAST, 2019), with S representing susceptible (standard dosing regimen), I susceptible (increased exposure), R resistant and UNK unknown [9]. When presenting results, isolates reported as UNK were not included in the denominator and isolates other than S were grouped in the NS (non-susceptible) category. In terms of reporting AMR, some microorganisms required specific antibiotic markers; oxacillin and glycopeptides for *Staphylococcus aureus*, glycopeptides for *Enterococcus* species, third-generation cephalosporins and carbapenems for Enterobacterales, and carbapenems for *Pseudomonas aeruginosa* and *Acinetobacter* species (spp.).

Two AMR indicators were estimated. First, the composite AMR index was calculated as the percentage of isolates with reduced or no susceptibility to first-level pathogen-specific antimicrobial markers; Methicillin-resistant *S. aureus* (MRSA), vancomycin-resistant Enterococci (VRE), Enterobacteriaceae non-susceptible to third-generation cephalosporin (3GC-NS) and carbapenem-resistant *P. aeruginosa* and *Acinetobacter* species. Only isolates with known susceptibility results were included. The second indicator was the percentage of Enterobacteriaceae that were not susceptible to carbapenems (CAR-NS) [7, 10].

Antimicrobial agents prescribed on the day of the survey were recorded. Additionally, antibiotics for surgical prophylaxis prescribed the day before the survey were recorded. Eligible antimicrobials were antibacterials and antifungals (2021 ATC codes; J01, J02, A07AA, P01AB, D01BA, J04AB02), based on the survey protocol [7]. Antimicrobials are presented as agents using the ATC5 codes and as classes using ATC4 codes [11].

Recording of AU indications (e.g., treatment of infection, prophylaxis) was based on prescriber’s documented aetiology. Antimicrobials prescribed for treatment were further specified with regards to the place of exposure (community, LTCF, or hospital-acquired) and diagnosis (e.g., pneumonia, cystic fibrosis, febrile neutropenia). For surgical prophylaxis, course duration i.e., a single dose, one day (multiple doses over one day), or more than one day (multiple doses over multiple days) was recorded.

To assess the utilization of broad-spectrum antibiotics two distinct indicators were used. The first indicator was derived from the 2021 WHO AWaRe (Access, Watch, Reserve) classification of antibiotics and was expressed as the percentage of antibiotics in each of the three AWaRe categories to the total number of antibiotics with an AWaRe designation [12]. The second was the percentage of broad-spectrum antibiotics to total antibiotics as defined by the ECDC, EFSA and EMA. Broad-spectrum antibiotics were piperacillin and enzyme inhibitor (J01CR05), third-generation cephalosporins (J01DD), fourth-generation cephalosporins (J01DE), monobactams (J01DF), carbapenems (J01DH), fluoroquinolones (J01MA), glycopeptides (J01XA), polymyxins (J01XB), linezolid (J01XX08) and daptomycin (J01XX09) [13].

### Data entry

Data from each hospital was recorded on-site by the data collection team and merged into a national database in the ECDC HelicsWin.Net software v.4.4.0 by the EODY team [14]. Data quality check was performed by EODY team with the use of the software, and invalid records were excluded. Each eligible patient received a unique identification number and no personal data was recorded. EODY is legally authorised by Greek law to process epidemiological data for public health purposes. The study was approved by the Institutional Ethics Review Board of EODY.

### Data analysis

The prevalence of HAI and AU was calculated as the percentage of hospitalised patients presenting at least one HAI and receiving at least one antimicrobial, respectively.

Descriptive analysis was performed to calculate central and dispersion measures. Distribution of variables was tested using the Kolmogorov-Smirnov test and 95% confidence intervals (CI) were calculated for mean in normally distributed variables or median in non-normally distributed variables using the bootstrapping method.

Hospital and ward exposure variables were tested for their possible association with HAI and AU prevalence. Descriptives are reported as absolute and relative frequencies for categorical variables and as mean (95% CI) or median (interquartile range, IQR) for continuous variables. Univariable analysis was performed with parametric tests (ANOVA with Bonferroni correction for multiple comparisons, student t-test, Pearson correlation coefficient) when the assumption of normality was met or with nonparametric tests otherwise (Mann-Whitney U test, Spearman’s rank correlation coefficient). Multivariable linear regression was performed for each outcome including variables with a statistically significant association in the univariable analysis. Correlation coefficients (β) and standard errors (SE) were calculated. Statistical significance was set at the level of 5%. Statistical analysis was performed using the statistical software R 4.1.1 (R Core Team, 2021) and RStudio 1.3.1093 package.

## Results

Overall, 50 hospitals (39.7% of Greek hospitals) were included from 12 out of 13 NUTS-2 level country regions; 25 (50%) secondary hospitals, 21 (42%) tertiary and 4 (8%) paediatric. Two hospitals denied participation and were replaced. A total of 9,707 hospitalised patients were included in the analysis.

### Healthcare-associated infections

Among the 9,707 inpatients, 1,175 had at least one HAI resulting in a prevalence of 12.1%. HAI prevalence was 14.3% in tertiary hospitals, 7.5% in secondary hospitals and 3.5% in paediatric hospitals. On average, 1.2 infections (range 1 to 4) were reported for each infected patient.

The highest prevalence was recorded in intensive care units (ICU) where 45.7% of patients had at least one HAI. Medical and surgical specialties followed with a prevalence of 13.5% and 8.2%, respectively. HAI prevalence was lower among patients in paediatric and neonatal specialties (3%), obstetrics and gynaecology (1.4%), and psychiatry (1.2%).

Of the 1,408 HAIs, 69.6% (*n* = 980) occurred during current hospitalisation, 28.6% (*n* = 403) were already present on admission and in 1.8% (*n* = 25) the origin was unknown. Among the infections present on admission, 26.3% (*n* = 106) originated from the same hospital (the patient was readmitted), 31.2% (*n* = 126) from another hospital, 10.1% (*n* = 41) from LTCFs and the rest had an unspecified origin.

Lower respiratory tract infections (LRTI) accounted for 28.9% (*n* = 407) of all HAIs, most of which were cases of pneumonia (*n* = 351, 24.9% of total infections). Bloodstream infections (BSI) were the second most reported HAI type (*n* = 282, 20%), among which 4.6% (*n* = 65) were catheter-related. Urinary tract infections (UTI) followed (*n* = 184, 13.1%) (Table 1).

**Table 1:** Distribution of Healthcare-associated infections (HAIs) by infection type

Infection type	HAIs (N)	HAIs (%)
Pneumonia/LRT	407	28.9
Bloodstream^1^	282	20.0
Urinary tract	184	13.1
SARS-CoV-2 infection	118	8.4
Gastrointestinal^2^	104	7.4
Surgical Site	103	7.3
Systemic^3^	76	5.4
Skin/Soft tissue	59	4.2
Other/Unspecified	75	5.3
Total HAIs	1,408	100.0

^1^including catheter-related infections: 65/1,408 (4.6%)

^2^including *Clostridioides difficile* infections: 57/1,408 (4.0%)

^3^including clinically suspected sepsis: 61/1,408 (4.3%)

LRT, lower respiratory tract; HAIs, healthcare-associated infections

SARS-CoV-2 infections accounted for 8.4% of HAIs (*n* = 118) and most of them were assessed as of mild or moderate severity (*n* = 61, 51.7%) followed by increased severity (*n* = 48, 40.7% of SARS-CoV-2 infections), with asymptomatic infections being the least reported (*n* = 9, 7.6%).

Device-associated infections varied by HAI type. A vascular catheter was present in 61.6% of BSI that reported device presence (*n* = 172/279), intubation was reported in 38.3% (*n* = 129/337) of pneumonia cases and a urinary catheter in 77.1% (*n* = 135/175) of UTIs.

### Microorganisms

In 58.9% (*n* = 830/1,408) of HAI at least one microorganism was identified (in total, 1,259 isolated microorganisms). Bacteria comprised most of the identified pathogens (*n* = 1,053, 83.6%), followed by fungi (*n* = 115, 9.1%) and viruses (*n* = 91, 7.2%).

Gram-negative bacteria were most often reported (*n* = 735, 58.4% of total isolates). Bacterial isolates belonged most often to *Klebsiella* spp. (*n* = 258, 20.5% of all isolates), *Acinetobacter* spp. (*n* = 161, 12.8%) and *P. aeruginosa* (*n* = 128, 10.2%). Gram-positive bacteria accounted for 24.9% of isolates (*n* = 313), commonly identified as *Staphylococcus aureus* (*n* = 79, 6.3%), *Enterococcus* spp. (*n* = 78, 6.2%) and coagulase negative Staphylococci (*n* = 77, 6.1%). Among fungi, *Candida* spp. (*n* = 99, 7.9%) were most frequently identified.

Susceptibility results were available in 93.9% of bacterial isolates. Results for specific markers were available in 95.1% of them included in the composite AMR index and in 93.4% of Enterobacteriaceae. The AMR composite index was 69.3%. Enterobacteriaceae were found to be non-susceptible to carbapenems in 46.8% (Table 2).

**Table 2:** Antimicrobial resistance per selected microorganism-antimicrobial combinations

Microorganism	N tested with results*	N non-susceptible	Non-susceptible, %
*Staphylococcus aureus* / MRSA	39	21	53.8
**Enterococci, GLY-R**	**68**	**35**	**51.5**
*Enterococcus faecalis*	21	5	23.8
*Enterococcus faecium*	41	27	65.9
**Enterobacteriaceae, 3GC-NS**	**205**	**125**	**61.0**
*Escherichia coli* / 3GC-NS	30	6	20.0
*Klebsiella* spp. / 3GC-NS	134	105	78.4
*Enterobacter* spp. / 3GC-NS	12	3	25.0
**Enterobacteriaceae, CAR-NS**	**171**	**80**	**46.8**
*Escherichia coli* / CAR-NS	24	2	8.3
*Klebsiella* spp. / CAR-NS	114	73	64.0
*Enterobacter* spp. / CAR-NS	11	1	9.1
***Pseudomonas aeruginosa***, **CAR-NS**	**121**	**79**	**65.3**
***Acinetobacter baumannii***, **CAR-NS**	**150**	**143**	**95.3**

*Isolates are shown only if more than 10 microorganisms in each group were reported

MRSA, methicillin-resistant *Staphylococcus aureus*; GLY, glycopeptides; VRE, vancomycin-resistant Enterococci; 3GC, third-generation cephalosporins; CAR, carbapenems; NS, non-susceptible to standard dosing

### Antimicrobial use

AU prevalence was overall 55.4% (*n* = 5,376/9,707); 64.9% in tertiary, 53.5% in secondary and 39.5% in paediatric hospitals. Each patient on antimicrobial therapy received on average 1.7 antimicrobials. Among treated patients, 51.9% (*n* = 2,788) received one antimicrobial, 35.1% (*n* = 1,890) received two and 13% (*n* = 698) received at least three.

AU prevalence was highest in ICU in 71.3% (*n* = 429/602). Among patients of surgical and medical specialties, AU prevalence was 59.4% (*n* = 1,781/2,999) and 58.2% (2,649/4,548), respectively.

**Fig. 1:**
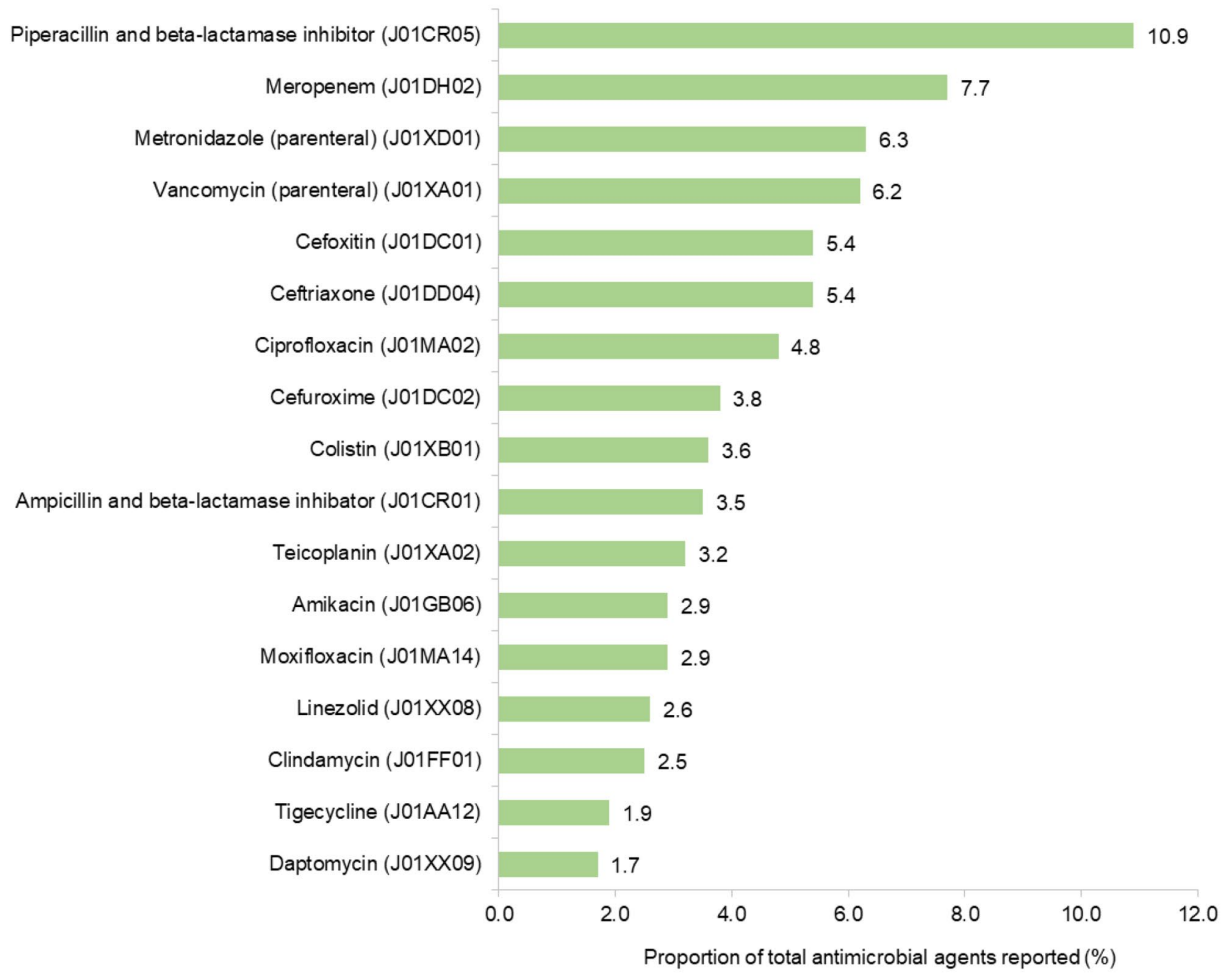
Antimicrobial agents (ATC5 code) accounting for 75% of study antimicrobial use

Overall, 9,003 antimicrobials were recorded. Antimicrobial agents belonged most frequently to the class of penicillin combinations including beta-lactamase inhibitors in 16.2% (*n* = 1,460) and followed by second-generation cephalosporins in 10.9% (*n* = 978), glycopeptides in 9.4% (*n* = 847), fluoroquinolones in 9.2% (*n* = 827) and carbapenems in 8.3% (*n* = 748). Most frequently recorded antimicrobial agents, accounting for 75% of total AU are presented in Fig. 1.

Antimicrobials were intended for treatment of community-acquired infections in 34.6% of all prescriptions (*n* = 3,114), hospital-acquired infections in 22.9% (*n* = 2,062) and LTCF-acquired infections in 5.1% (*n* = 460). Prophylactic use followed with 10.9% (*n* = 983) prescribed for medical conditions and 18.4% (*n* = 1,656) for surgical procedures. The indication was other or unknown in 5.6% (*n* = 502) and 2.5% (*n* = 226) of antimicrobials, respectively.

**Fig. 2:**
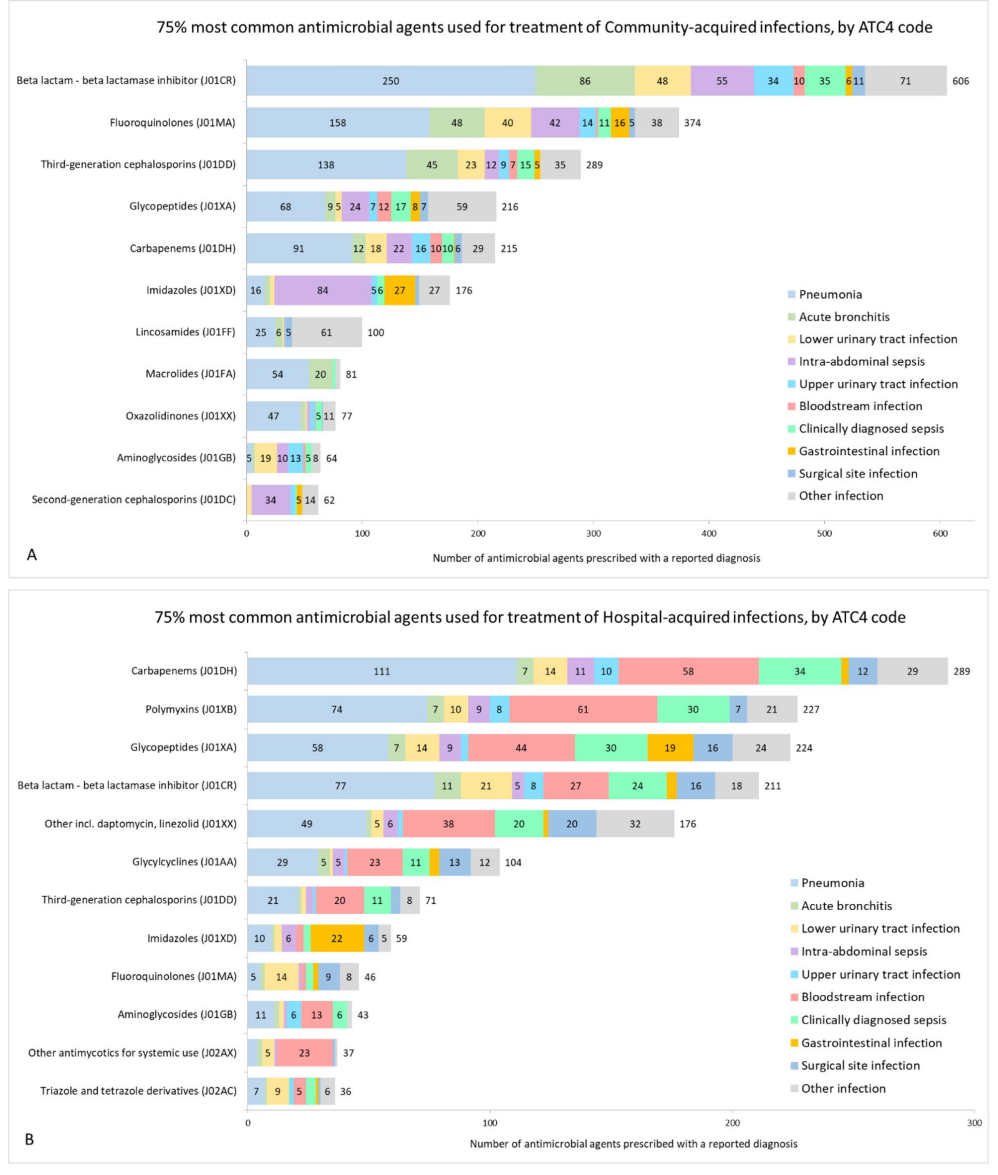
The 75% most frequent antimicrobial classes (ATC4 code) indicated for treatment of Community-acquired (**A**) and Hospital-acquired (**B**) infections, by most commonly reported diagnoses. Values below 5 are not shown

Diagnosis for antimicrobials prescribed for treatment was provided in 97.9% of the cases (*n* = 5,520/5,636). The most frequent antimicrobial classes (75% of total) by infection origin and reported diagnoses are presented in Fig. 2. Absolute frequencies are shown for the most common (75%) ones in each class and the rest of the diagnoses are grouped in the category “other”. The most frequent antimicrobial classes were carbapenems for treatment of hospital infections accounting for 14.3% (*n* = 289/2,025) and beta lactam combinations for community-acquired infections in 19.9% (606/3,039).

### Surgical prophylaxis

**Fig. 3:**
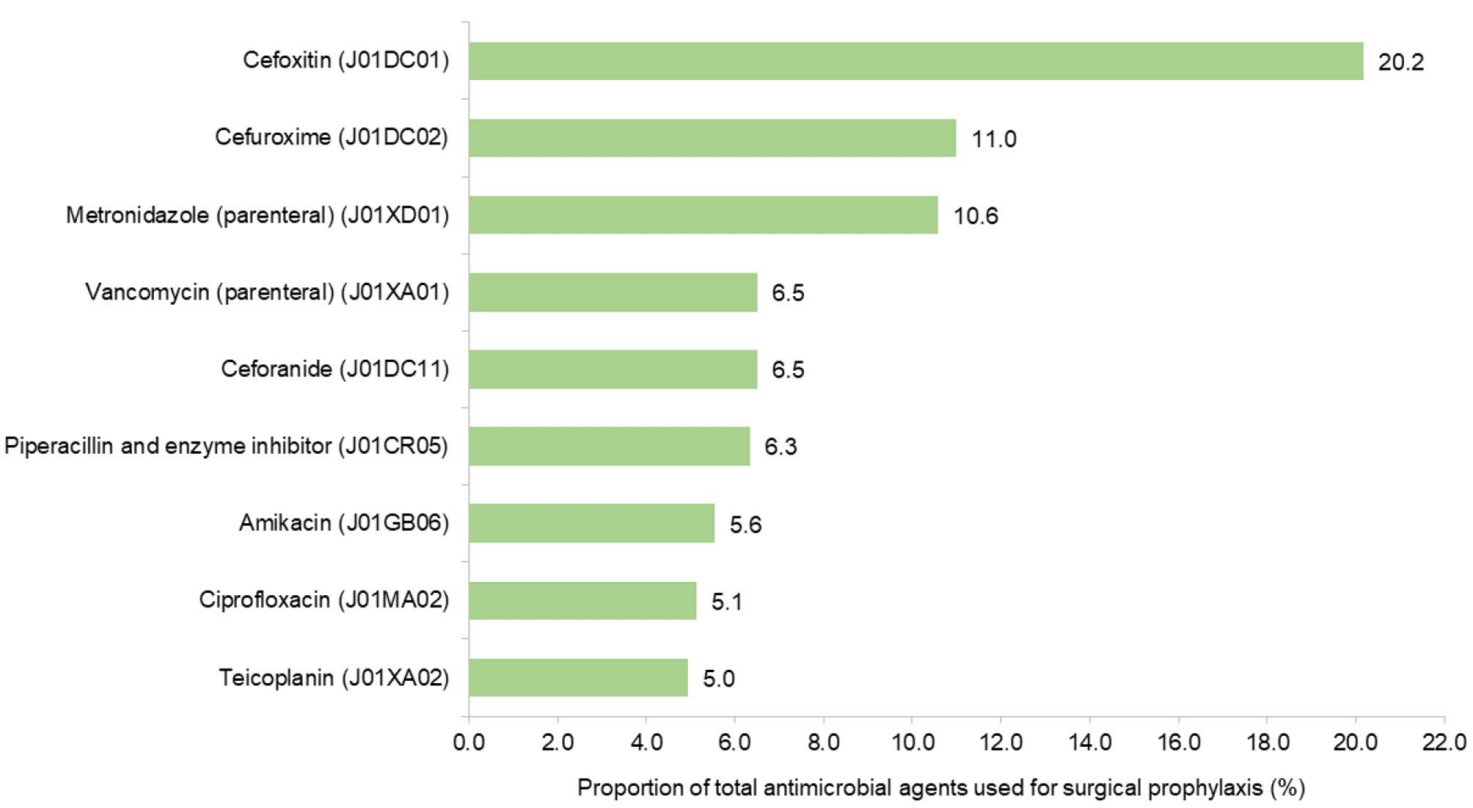
Frequent antimicrobial agents (ATC5 code) accounting for 75% of surgical prophylaxis indication

As for surgical prophylaxis prescriptions (*n* = 1,656), a single dose was given in 5.8% of cases (*n* = 96), one-day courses in 18.5% of them (*n* = 307) and courses lasting more than one day in 75.7% (*n* = 1,253). Surgical prophylaxis antimicrobials (*n* = 1,656) belonged most frequently to second-generation cephalosporins in 38.6% (*n* = 645) and were followed by glycopeptides in 11.4% (*n* = 190), imidazoles in 10.5% (*n* = 175), combinations of penicillins including beta-lactamase inhibitor in 9.7% (*n* = 162) and fluoroquinolones in 5.7% (*n* = 95). The antimicrobial agents that were used for surgical prophylaxis are presented in Fig. 3, ranked by frequency.

### Spectrum of antimicrobial coverage

Of 9,003 antimicrobials recorded, 8,567 agents were antibacterials (95.2%) and 8,508 were classified in the AWaRe list (94.5% of total). Broad-spectrum antibacterials comprised 58.1% of antibacterials (4,979/8,567), and the ratio of broad-spectrum to narrow-spectrum antibiotics was 1.4. Prescribed agents came from the restricted classes of Watch in 63.4% (*n* = 5,397/8,508) and Reserve group in 13.3% (*n* = 1,127/8,508).

### Risk factor analysis

Among the 50 sample hospitals, the median HAI prevalence was 8.9% (95% CI: 5.5–10.5) with 25% and 75% percentiles of 3.5% and 12.4%, respectively. Descriptive statistics of independent and outcome variables assessed are presented in Supplementary Table 1 (ST1).

Results from univariable and multivariable linear regression analysis are presented in Supplementary Table 2 (ST2) and Table 3, respectively. After adjusting for covariates, HAI prevalence presented a statistically significant positive association with bed occupancy (β = 0.107, SE = 0.046, *p* = 0.027). Higher AU prevalence was associated with the secondary hospital type (β = 19.98, SE = 7.838, *p* = 0.014) in the multivariable analysis.

**Table 3:** Results from the multivariable linear regression analysis of hospital factors associated with antimicrobial use (AU) and healthcare-associated infections (HAI).

Variables	AU prevalence (per 1% increase)			HAI prevalence (per 1% increase)		
	β coefficient	SE	*p*-value	β coefficient	SE	*p*-value
Hospital size (total beds)	0.003	0.014	0.825	0.008	0.004	0.060
Bed occupancy, %	-0.225	0.158	0.163	0.107	0.046	**0.027**
Total beds per 1 ID doctor	-	-	-	0.002	0.005	0.617
Current surgical patients per total inpatients, %	0.216	0.142	0.135	-	-	-
Hospital type^1^ (secondary)	19.980	7.838	**0.014**	-	-	-
Hospital type^1^ (tertiary)	12.168	8.726	0.170	-	-	-

^1^peadiatric hospitals were used as the reference category for hospital type

AU, antimicrobial use; HAI, healthcare-associated infections; ID, infectious disease; SE, standard error

## Discussion

PPS is an important tool to depict the burden of infections in hospital settings and to compare the country-specific changes in time through representative HAI, AMR, and AU indices.

Based on the 2022 PPS in Greece, a possible upward trend in HAI prevalence is discerned; 12.1% of hospitalised patients had at least one HAI episode in 2022, while the respective percentage was 10.0% in 2016–2017 and 9.0% in 2011–2012 [6, 10, 15, 16]. In the first European PPS in 2011–2012, HAI prevalence was 6.0% (country range 2.3 − 10.8%) with Greece recording the fourth highest HAI prevalence [16]. In the following PPS in 2016–2017, the European prevalence was similar, at 5.9% (country range 2.9 − 10.0%) but Greece reported the highest prevalence [6, 10].

As in the previous Greek PPS, the most frequently reported HAI was LRTI, comprising a quarter of HAI cases, with BSI and UTI following. In the European PPS, although LRTIs were also the most common HAI type, a less severe infection -UTI- followed, while BSI were almost half of those reported in the Greek sample [6, 15, 16]. SARS-CoV-2 infections, included for the first time in the protocol, were fourth in frequency and in 41% of cases were assessed as of increased severity, which is partially attributed to the vulnerable condition of inpatients [17].

SSIs were reported less frequently than the last Greek and European PPS [6]. A possible explanation is the requirement of retrospective data collection in SSI cases. Attributing current HAI to a past operation was challenging due to the lack of a national reporting system on surgical operations that could be accessed by data collectors; recording relied on patient-provided data. Nevertheless, PPS is a valuable reporting system for SSIs that provides standardised data on these infections. Reporting of gastro-intestinal infections has similar findings with the previous PPS with *C. difficile* infections steadily accounting for almost half of the recorded cases, possibly explained by the observed stable rate of antibiotic exposure, an established risk factor for *C. difficile* infection [18].

With regards to isolated microorganisms, Gram-negative bacteria comprised the majority, as expected in a hospital setting, similar to previous studies [6]. Difficult-to-treat pathogens were common in this survey in line with the previous two PPS in Greece [6, 15, 16]. Approximately 54% of *Staphylococcus aureus* isolates were methicillin-resistant, similar to a 50% rate reported in 2011–2012 [15]; this is somewhat higher than previous community reports from the Greek AMR surveillance network in 2021 that estimated a 41.9% rate of MRSA [19]. These percentages are in stark contrast to the European MRSA rate of 15.8% in both hospital and community isolates [19].

Resistance to first-level antibiotics was reported in 69.3% of selected isolates, similar to the 2016 Greek PPS results (61.2%) and to data reported in the European Antimicrobial Resistance Surveillance Network (EARS-Net) in 2021 at 61.3% [10, 19]. In EARS-Net data, only *K. pneumoniae* and *E. coli* are reported from the Enterobacteriaceae family and thus, could be considered for the index calculation. The composite index is steadily higher than the 31.6% reported on average by European countries in 2016–2017 [10]. As for the second AMR index, it lies between the 43.7% recorded in the last PPS and the 49.1% reported in EARS-Net 2021 resistance data in Greece [10, 19]. Again, these results differ substantially from the European average rate of 6.2% [10]. In particular, *Klebsiella* isolates with carbapenem resistance mechanisms were still frequent; 64% compared to 67% in the previous study [6].

Antimicrobial use remained high in Greece with over half of hospitalised patients receiving at least one antimicrobial agent. In fact, rates of use remain stable and essentially unchanged over the last decade; AU prevalence was 55.4% in 2022, 55.6% in 2016–2017 and 54.7% in 2011–2012 [6, 16]. Greece held the first place among participating European countries in both previous studies, while the EU/EEA average was 35.0% (range 21.4–54.7%) in 2011–2012 and 32.9% (range 15.9–55.6%) in 2016–2017 [16, 20]..

Importantly, antimicrobials used had more commonly a broad-spectrum coverage than a narrow one, at a ratio of 1.4. Piperacillin-tazobactam and meropenem were the two most commonly prescribed agents for all indications. WHO suggests that at least 60% of antibiotic consumption at the national level be from the Access group which includes antibiotics that have a narrow spectrum of activity, low resistance and good safety profile compared to antibiotics in Watch and Reserve groups that are intended for more severe clinical presentations or resistant pathogens [12, 21]. In this survey, one-fifth of administered antibiotics came from the Access group, while the overall choice of agents was shifted to broader spectrum antimicrobials with more than 60% coming from the Watch group.

As for surgical prophylaxis, cefoxitin and cefuroxime, two second-generation cephalosporins, were the most frequently prescribed agents. The recorded antibiotics are consistent with 2022 guidelines issued by professional societies in Greece, where second-generation cephalosporins -and vancomycin in certain types of procedures- are recommended, and emphasis is placed on single-dose antimicrobial prophylaxis perioperatively for most procedures [22]. The antimicrobial spectrum of guideline-directed agents is broader than that of antibiotics proposed in other parts of Europe and the US, where the first-line treatment for most procedures is first-generation cephalosporins, such as cefazolin, and clindamycin as an alternative. Longer courses are to be used in interventions involving active infections or contaminated sites [23, 24]. Although these procedures are not so common, most patients in this study received courses of treatment instead of prophylaxis, which is an alarming finding and in stark contrast to international recommendations [25, 26]. Antimicrobial overuse in this group of patients should be an important primary focus of antimicrobial stewardship interventions.

In this study, several factors possibly associated with HAI and AU prevalence at the hospital level were assessed individually. After adjusting for covariates, increasing bed occupancy in the hospital showed a positive association with HAI prevalence. This relation could possibly be attributed to IPC-related concerns, such as staff shortages, difficulties in case isolation and hygiene compliance as well as increased workload of healthcare workers that puts a strain on optimal patient care. After taking into account all covariates, secondary hospitals showed increased AU prevalence. It is our understanding that these hospitals in Greece, which are mostly situated in regional settings, may often lack diagnostic and interventional resources as well as robust antimicrobial stewardship programs compared to tertiary and specialised hospitals [27, 28].

The main limitation of this study is its cross-sectional design, in which HAI and AU burden is conveyed as a “photographic” depiction in time. Longitudinal studies or prospective surveillance programs could elucidate the true burden of HAI and identify patients at risk. Regarding the presence of invasive devices relevant to a specific infection, it was not possible to discern a potentially causal role, except for a limited number of intravascular catheter-related cases. A deep dive into local clinical data with the assistance of the treating physician is necessary for such an analysis. The high variance observed in prevalence between sample hospitals could be traced partly to difficulties involved in the identification of HAI cases which was more complex than AU and relied on the availability of dedicated data collectors with surveillance knowledge and time. Another limitation of the study is the absence of clarifying information in the AU indication section regarding the type of surgical procedure that a prophylaxis was selected for. Thus, the link between clean or contaminated surgical intervention and respective optimal surgical prophylaxis remains to be elucidated in further studies.

## Conclusion

The 2022 PPS study highlights the significant challenge that HAIs pose in patient care in Greece. It clearly indicates an increased burden of HAIs together with the emergence of difficult-to-treat pathogens in inpatients, as well as the extensive use of broad-spectrum antimicrobials. Subsequent studies will provide additional evidence regarding the trend in HAI prevalence and prescription attitudes and support the identification of modifiable practices. The strengthening of IPC and AMS programs in these settings is essential.

### Electronic supplementary material

Below is the link to the electronic supplementary material.

Supplementary Material 1

## Data Availability

The data that support the findings of this study are available from the National Public Health Organization (EODY) but restrictions apply to the availability of these data, which were used under license for the current study, and so are not publicly available. Data are however available from the authors upon reasonable request and with permission of the National Public Health Organization (EODY).
