# Supplementary material for: Healthcare-associated infections and antimicrobial use in acute care hospitals in Greece, 2022; results of the third point prevalence survey
Source: Antimicrob Resist Infect Control. 2024 Jan 25;13:11. doi: 10.1186/s13756-024-01367-8 (PMC10809483; doi:10.1186/s13756-024-01367-8)
Supplement: Supplementary file 1 — Supplementary Material 1 [file 13756_2024_1367_MOESM1_ESM.docx]

**Supplementary material**

**Supplementary Table 1 (ST1)**: Descriptive statistics of the variables included in the study analysis.

| ST1. Descriptive statistics of the variables included in the study analysis. | | |
| --- | --- | --- |
| Variable groups | **Variables** | **Median (25th, 75th percentile) or**  **n/N (frequency, %)** |
| Hospital characteristics | Hospital size (total beds) | 319 (172, 528) |
|  | Hospital type |  |
|  | paediatric | 4/50 (8.0) |
|  | secondary | 25/50 (50.0) |
|  | tertiary | 21/50 (42.0) |
| Workload indicators | Bed occupancy, % | 54.4 (45.6, 67.9) |
|  | Current surgical patients per total inpatients, % | 31.8 (26.2, 40) |
|  | Healthcare workers per current inpatients | 1 (1, 2) |
| IPC and AMS indicators | Total beds per 1 ID doctor^1^ | 0 (0, 185) |
|  | Healthcare workers vaccinated for influenza^2^, % | 29.7 (20.5, 43.1) |
| COVID-19 burden | Current COVID-19 cases per total inpatients, % | 5.6 (1.9, 9.6) |
|  | Healthcare workers vaccinated for COVID-19^2^, % | 93.5 (87, 96.6) |
| Outcome variables | AU prevalence | 59.5 (50.9, 68.6) |
|  | HAI prevalence | 8.9 (3.5, 12.4) |
| ^1^cases with 0 values correspond to hospitals that recorded 0 number of full-time equivalent infection control doctors  ^2^data refer to 2021.  Missing values were excluded.  IPC, infection prevention and control; AMS, antimicrobial stewardship; AU, antimicrobial use; HAI, healthcare-associated infections; ID, infectious disease | | |

**Supplementary Table 2 (ST2)**: Results from the univariable correlation analyses of hospital factors potentially associated with antimicrobial use (AU) and healthcare-associated infections (HAI).

| ST2. Results from the univariable correlation analyses of hospital factors potentially associated with antimicrobial use (AU) and healthcare-associated infections (HAI). | | | | | |
| --- | --- | --- | --- | --- | --- |
|  | | **AU prevalence** | | **HAI prevalence** | |
| Exploratory variables | **N** | **Correlation coefficient** | **p-value** | **Correlation coefficient** | **p-value** |
| Hospital size (total beds) | 50 | -0.311 | **0.028** | 0.580 | **0.000** |
| Bed occupancy, % | 49 | -0.408 | **0.004** | 0.540 | **0.000** |
| Total beds per 1 ID doctor | 40 | -0.217 | 0.178 | 0.386 | **0.014** |
| Current COVID-19 cases per total inpatients, % | 39 | 0.202 | 0.217 | 0.214 | 0.190 |
| Current surgical patients per total inpatients, % | 50 | 0.300 | **0.034** | -0.153 | 0.290 |
| Healthcare workers vaccinated for COVID-19^1^, % | 44 | 0.161 | 0.296 | -0.024 | 0.877 |
| Healthcare workers vaccinated for influenza^1^, % | 44 | -0.280 | 0.066 | 0.003 | 0.985 |
| Healthcare workers per current inpatients | 34 | 0.142 | 0.422 | -0.037 | 0.835 |
| Hospital type | 50 |  |  |  |  |
| paediatric | 4 | ref. |  | ref. |  |
| secondary | 25 | 23.387 | **0.015** | 4.751 | 1.000 |
| tertiary | 21 | 11.713 | 0.455 | 10.404 | 0.182 |
| ^1^data refer to 2021.  AU, antimicrobial use; HAI, healthcare-associated infections; ID, infectious disease | | | | | |
